# Efficacy of DiscoGel in Treatment of Degenerative Disc Disease: A Prospective 1-Year Observation of 67 Patients

**DOI:** 10.3390/brainsci11111434

**Published:** 2021-10-28

**Authors:** Kajetan Latka, Klaudia Kozlowska, Marek Waligora, Waldemar Kolodziej, Tomasz Olbrycht, Jacek Chowaniec, Stanislaw Hendryk, Miroslaw Latka, Dariusz Latka

**Affiliations:** 1 Department of Neurosurgery, University Hospital in Opole, Institute of Medicine, University of Opole, 45-001 Opole, Poland; wkolodziej@mp.pl (W.K.); tomaszolbrycht@gmail.com (T.O.); jacekchowaniec2015@gmail.com (J.C.); dlatka@mp.pl (D.L.); 2 Center for Minimally Invasive Spine and Peripheral Nerves Surgery Latka and Partners, 45-064 Opole, Poland; 3 Department of Biomedical Engineering, Wroclaw University of Science and Technology, 50-370 Wroclaw, Poland; klaudia.kozlowska@pwr.edu.pl (K.K.); miroslaw.latka@pwr.edu.pl (M.L.); 4 Clinical Department of Diagnostic Imaging, Faculty of Medicine, University of Opole, 45-040 Opole, Poland; marekwaligoraluxmed@gmail.com; 5 Medical University of Silesia, 40-055 Katowice, Poland; stanhendryk@interia.pl

**Keywords:** chemonucleolysis, discogenic pain, ethanol gel, low back pain, percutaneous spine surgery, radiculopathy

## Abstract

Patients with degenerative disc disease may suffer from chronic lumbar discogenic (DP) or radicular leg (RLP) pain. Minimally invasive DiscoGel therapy involves the percutaneous injection of an ethanol gel into the degenerated disk’s nucleus pulposus. This paper compares the 1-year outcome of such treatment in DP and RLP patients. We operated on 67 patients (49 men and 18 women) aged 20–68 years (mean age 46 ± 11 years) with DP (*n* = 45) and RLP (*n* = 22), of at least 6–8 weeks duration, with no adverse effects. We evaluated the treatment outcome with Core Outcome Measures Index (COMI) and Visual Analog Scale (VAS). A year after the ethanol gel injection, in the DP cohort, COMI and VAS dropped by 66% (6.40 vs. 2.20) and 53% (6.33 vs. 2.97), respectively. For the RLP patients, the corresponding values dropped 48% (7.05 vs. 3.68) and 54% (6.77 vs. 3.13). There were no differences between the cohorts in COMI and VAS at the follow-up end. Six months into the study, 74% of DP and 81% of RLP patients did not use any analgesics. Ethanol gel therapy can be effective for many patients. Moreover, its potential failure does not exclude surgical treatment options.

## 1. Introduction

Intervertebral discs (IVDs) are indispensable for the normal functioning of the human spine. They act as ligaments that hold the adjacent vertebrae together, shock absorbers, and “pivots” allowing the spine to bend, rotate, and twist. The IVD consists of the nucleus pulposus (NP), a water-rich gelatinous center that primarily bears pressure; the annulus fibrosus (AF), a fibrous structure of 15–25 concentric sheets of collagen (lamellae) that confines the pressurized NP; and the vertebral end-plates (VEP), which are cartilaginous plates that are interwoven into the AF at the disc-vertebrae interface and supply nutrients to the disc. The high concentration of negatively charged proteoglycans in NP helps retain water and maintain NP swelling pressure [1].

Advanced IVD degeneration is characterized by disc height loss, osteophyte formation, intranuclear calcification, VEP sclerosis, and AF fissures or tears [2,3].

Patients with degenerative disc disease (DDD) may primarily report discogenic pain (DP) or radicular leg pain (RLP) that belongs to a broad class of low back pain (LBP) [4]. RLP related to disc degeneration may radiate from the back down the leg along the distribution of a specific nerve root or more diffusely [5,6]. DP is associated with the IVD degeneration without herniation or anatomical deformity. This kind of LBP is caused by multiple factors, and its pathophysiology is not fully understood [3]. It usually requires prolonged treatment and has mixed-to-poor surgical outcomes [7].

In the normal IVD, sensory (mainly nociceptive), postganglionic sympathetic, and sinuvertebral nerves innervate the outermost third of the AF. Deep AF fissures that extend radially from the NP can stimulate nerve fibers and produce pain. [8]. There is evidence that DDD leads to nerve ingrowth into the inner AF layers [9,10,11], which in turn can sensitize IVD to even normal mechanical loads. Inflammatory cytokines can penetrate NP through VEP fractures. The resulting inflammation response affects oxygen diffusion, pH, and lactate concentration. In some cases, the cytokines themselves can be the source of pain. Thus, both mechanical and chemical factors can lead to IVD sensitization [8].

The risk of severe and chronic LBP increases with age [12,13]. Meucci et al., estimated chronic LBP prevalence at 4.2% in individuals aged between 24 and 39 years old and 19.6% in those aged between 20 and 59 [14]. It is worth mentioning that LBP can even affect children and adolescents [15] as well as pregnant women [16]. Using lumbar discography, Verrills et al., estimated the prevalence of discogenic pain in LBP cohort at 21.8% (95% CI: 17–26%) [17]. The incidence of radicular symptoms in patients presenting with low back pain ranges from 12% to 40% [18].

LBP patients suffer not only from musculoskeletal and neuropathic pain conditions but also from its sequelae (e.g., depression, anxiety, and sleep disorders) [19]. Prolonged work disability and high direct medical costs impose a substantial burden on healthcare systems. The Global Burden of Disease Study (GBD) ranked LBP as one of the leading causes of years living with disability [20,21,22].

There are conservative and invasive LBP treatment strategies. The former combines pharmacotherapy and physical therapy and is particularly important for patients who are not good surgical candidates [23]. The latter include disc excision with laminectomy, microdiscectomy, spinal fusion, and artificial disc replacement. Considerable morbidity and long recuperation of conventional surgical methods led to the development of minimally invasive procedures. Percutaneous lumbar disc access enables thermal, chemical, or mechanical decompression in an outpatient setting with minimal disruption of the surrounding tissues [24].

In 1964, Lyman Smith injected chymopapain [25] into the IVD to dissolve the herniated NP. This percutaneous chemonucleolysis was used in the United States for about 20 years until its demise, which was caused by the combination of medical (allergic and other adverse reactions) and political factors [26]. In 2007, Theron et al., proposed an ethanol gel as an alternative to chymopapain [27]. It is a sterile viscous solution containing ethyl alcohol, cellulose derivative product, and tungsten. The presence of cellulose, a gelling agent, limits the risk of epidural leaks that may occur with pure ethanol. The mechanisms of action of ethanol gel may include intradiscal pressure reduction (brought about by NP dehydration), lytic action on nerve ingrowth, and local NP necrosis. Ethanol gel is easy to handle, and the presence of radiopaque tungsten facilitates injection monitoring. Several studies demonstrated the efficacy and safety of gelified ethanol injection (GEI) [28,29,30,31,32]. Please note that in the literature this procedure is also referred to as ethanol gel chemonucleolysis.

While percutaneous decompression techniques are effective in alleviating radicular lumbar pain [33], GEI efficacy in discogenic pain treatment has not been thoroughly studied [34,35]. This paper compares the 1-year outcome of GEI treatment of patients with radiculopathy and discogenic pain.

## 2. Materials and Methods

### 2.1. Study Design and Patient Recruitment

In this prospective uncontrolled clinical study, the patients were allocated to radiculopathy and discogenic pain groups depending on the dominant nature of their symptoms. The RLP inclusion criteria were sciatica and irritative radicular symptoms (without motor deficits) associated with IVD protrusion/extrusion in spinal MRI. The patients with discogenic pain without positive radicular signs and with visible black disc lesions (with or without a high intensity zone) were assigned into the DP group.

### 2.2. Procedure

**Figure 1:**
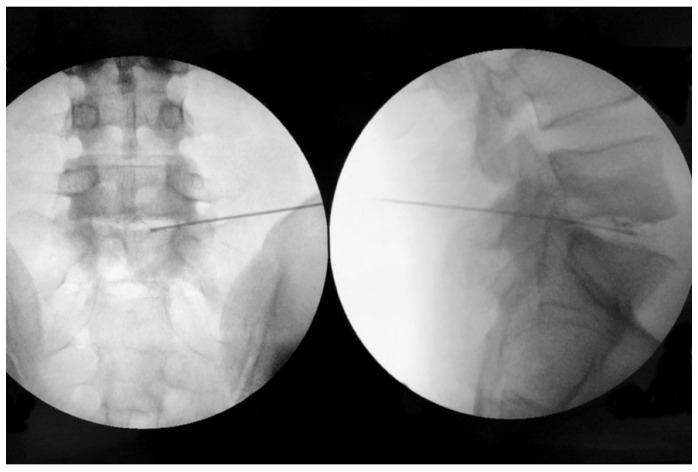
AP (**left**) and LAT (**right**) X-ray views of the ethanol gel injection under fluoroscopic guidance. The presence of tungsten in the gel provides a radiographic contrast effect as evidenced by contrast seen in the nucleus pulposus in the lateral view.

All GEIs were performed by the same operator (K.L.) in a day surgery unit with local subcutaneous anesthesia (2% lidocaine) without pre- or intra-operative antibiotic prophylaxis [36]. We used fluoroscopic guidance (Ziehm Solo X-ray C arm) to insert an 18G needle in the NP center (Figure 1). 0.9 mL of DiscoGel was injected directly through the needle at a rate of 0.1 mL/min. We removed the needle fifteen minutes after the injection was completed. We introduced this delay into the procedure as an ethanol gel leakage precaution. Total radiation exposure during the procedure was recorded. The patients were discharged after three-hour monitoring as suggested by the Discogel manufacturer. They were asked to refrain from physical work, sports, sudden spinal rotation, and bending for three weeks. None of them underwent rehabilitation.

### 2.3. Data Collection

At baseline and four follow-ups (F1: 4–8 weeks, F2: 12–24 weeks, F3: 24–36 weeks, F4: 36-52 weeks), we assessed the patient’s clinical and pain conditions using the Polish version [37] of Core Outcome Measures Index (COMI) [38] and Visual Analog Scale (VAS), respectively. The patients gave the VAS score for general, leg, and back pain. Six months after GEI, the following questionnaire was used to determine the consumption of analgesics:

- 0—I do not take any.
- 1—I take them once a week.
- 2—I take them several times a week.
- 3—I take them once a day.
- 4—I take them several times a day.

We asked blue-collar workers whether they perform light or heavy physical work (lifting and carrying loads heavier than 10 kg).

We measured the time after which the patients returned to work in weeks.

### 2.4. Statistical Analysis

The Shapiro–Wilk test was used to assess the normality of data. Differences in COMI and VAS scores between the RLP and DP cohorts at a given time point were assessed using Student’s *t*-test for independent samples or Mann–Whitney test. We used analysis of variance (ANOVA or Kruskal–Wallis test) to detect postoperative changes in the clinical parameters.

Pre- and post-treatment consumption of analgesics were compared using the Wilcoxon test. The same test was used to detect differences in time of return to work between the RLP and DP groups.

Statistical calculations were performed using the Python SciPy library. For all tests, the significance level was set to 0.05.

## 3. Results

### 3.1. Patient Characteristics

**Figure 2:**
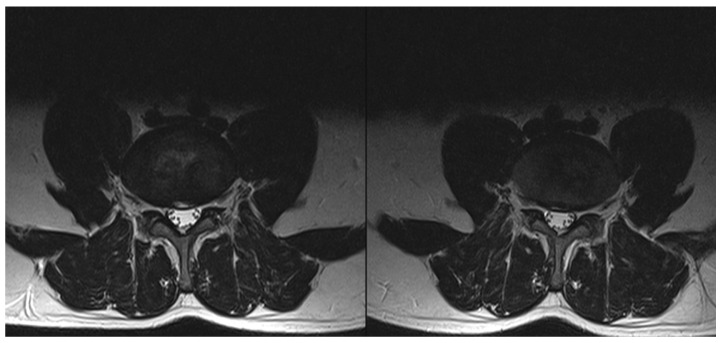
Pre- (**left**) and post-GEI (**right**) axial MRI at L4/L5 level of a patient with discogenic pain. MRI was performed two weeks before and six weeks after the GEI.

**Figure 3:**
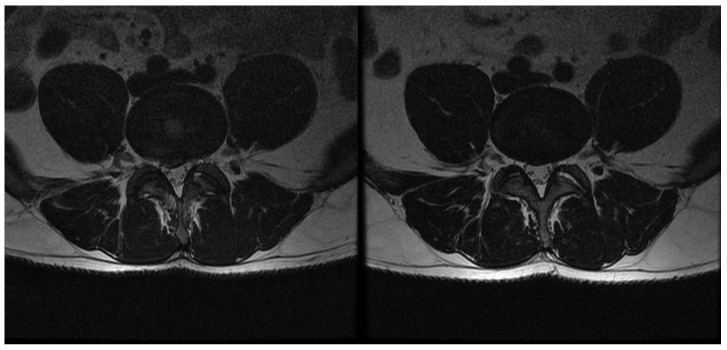
Pre- (**left**) and post-GEI (**right**) axial MRI at L4/L5 level of of patient with radiculopathy. MRI was performed four weeks before and six weeks after the GEI.

From October 2017 to August 2019, we enrolled 67 patients (49 men and 18 women) aged 20–68 years (mean age 46 ± 11 years) with DP (*n* = 45) and RLP (*n* = 22). Table 1 shows the patient characteristics. Twenty one of them performed heavy and 23 light physical work. The others were white-collar workers (*n* = 9) or unemployed (*n* = 14). The most frequently supplied level was L4/L5 (37 cases). We did not observe any adverse effects. In Figure 2 and Figure 3 we present examples of pre- and post-GEI MRI at L4/L5 level of DP and RLP patients, respectively.

**Figure 4:**
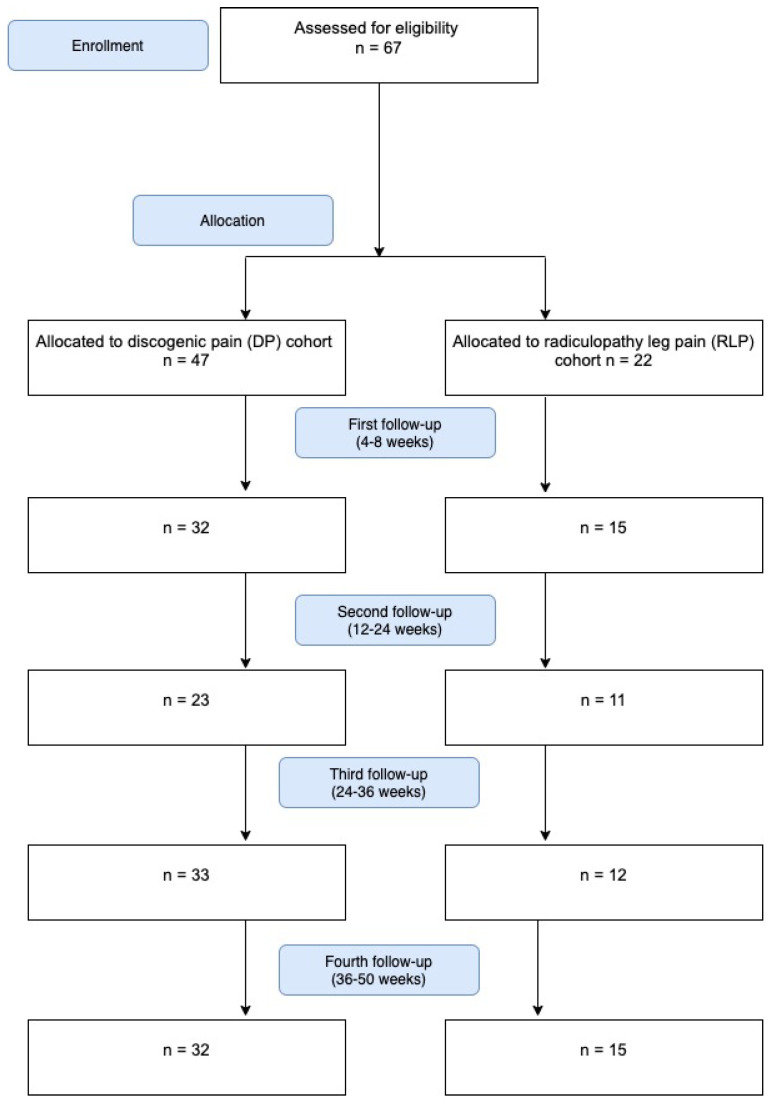
Flow chart of the recruitment and follow-up process.

Follow-up rates for both cohorts were about 70%. We lost contact with some of the non-Polish nationals. The patient flow is shown in Figure 4.

### 3.2. Follow-Up Evaluation

**Figure 5:**
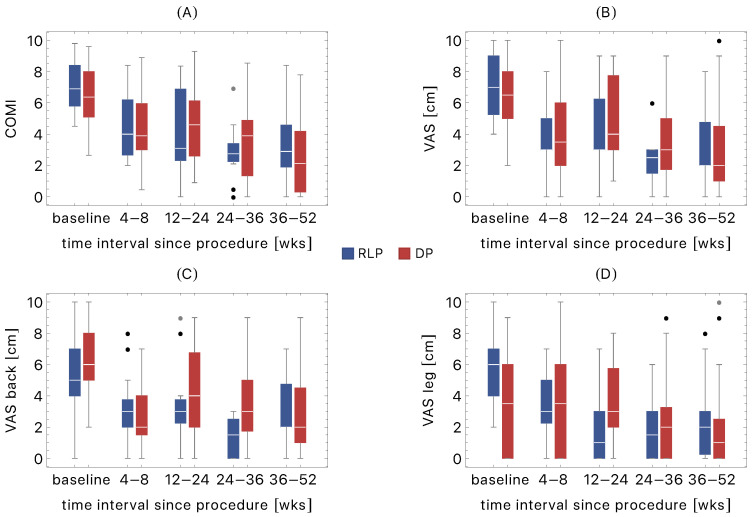
Boxplots of COMI (**A**), VAS (**B**), VAS back (**C**), and VAS leg (**D**) at baseline and four follow-ups: F1 (4–8 wks), F2 (12–24 wks), F3 (24–36 wks), and F4 (36–52 wks). The data are presented for radiculopathy (RLP) and discogenic pain (DP) groups. The black and grey dots correspond to the outliers and far outliers, respectively.

We collected the values of COMI, VAS, VAS back, and VAS leg at baseline and four consecutive follow-ups in Table 2. Figure 5 visualizes the distribution of all scores.

We observed the significant postoperative reduction of scores at all four follow-ups for:

- COMI (both cohorts)
- VAS (RLP cohort)
- VAS leg (RLP cohort)
- VAS back (DP cohort).

Except for F2, the same result was observed for DP VAS.

With respect to baseline, RLP VAS back decreased at the third and fourth follow-up. For DP group, the improvement of VAS leg occurred at F4.

### 3.3. Comparison of RLP and DP Cohorts

We observed no differences between the cohorts in COMI at baseline and follow-ups.

There were only two differences between the cohorts in pain assessment:

- The DP VAS back at F3 was higher (*p* = 0.02).
- The RLP VAS was higher at baseline (*p* = 0.001).

The time after which the DP and RLP patients returned to work was not different (4 weeks for RLP vs. 5 weeks for DP).

### 3.4. Consumption of Analgesics

Table 3 shows significant reduction of postoperative analgesic use. For both groups $p<1\times 10^{-4}$ (Wilcoxon signed rank test). We did not observe differences in consumption of analgesics between the groups (before and after the GEI).

## 4. Discussion

Various minimally invasive treatments have been proposed for chronic DP, such as intradiscal injections (dextrose solution, [39], platelet-rich plasma [40], bone marrow concentrate [41], lipoaspirate [42], ozone [43]), intradiscal electrothermal annuloplasty [44], automated percutaneous mechanical discectomy [45], and basivertebral/sinuvertebral nerve thermocoagulation [46].

In 2007, Theron et al., investigated the percutaneous treatment of lumbar IVD hernias with gelified ethanol [27]. The preliminary results have rekindled the interest in chemonucleolysis, which was abandoned about the turn of the century. In the ensuing years, several studies corroborated the ethanol gel efficacy in the treatment of radicular pain, sciatica, and disc hernias [28,29,30,31,32]. We are aware of only two papers on GEI in patients with lumbar DP [34,35].

In a 2014 pilot research, Papadopoulos et al., performed a combination of pulsed radiofrequency and GEI on a carefully selected DP cohort [34]. 11 enrolled patients suffered from chronic lower back pain, refractory to conservative treatment and physiotherapy. All reported pain during provocative discography. The procedure was fully successful in 9 cases (81.8%), as indicated by the pain and satisfaction scores. A follow-up, randomized, double-blind clinical study showed that both ethanol gel alone and ethanol gel in combination with pulsed radiofrequency reduced pain, consumption of analgesics, and improved function and quality of life. The effects persisted for 12 months. There were 18 patients in each treatment group [35].

To the best of our knowledge, we performed GEI on the largest (*n* = 45) DP cohort. COMI, VAS, and VAS back scores decreased at the first follow-up (4-8 weeks) by 28%, 36%, and 40%, respectively. At the end of the study, the corresponding declines were equal to 66% (6.40 vs. 2.20), 53% (6.33 vs. 2.97), and 71% (6.15 vs. 1.80). Six months after GEI, 74% of DP patients did not use any analgesics. On average, the employees returned to work after five weeks.

We observed similar treatment effects in RLP patients. In particular, there were no differences between the cohorts in COMI at baseline and all follow-ups. There were only two differences between the cohorts in pain assessment (Table 2). The final VAS and VAS leg was equal to 3.13 and 2.57, respectively. Eighty one percent of RLP patients did not use any analgesics six months into the study. They returned to work a week earlier than the DP cohort, but the difference was not statistically significant. The research groups which used VAS to assess the GEI induced pain changes in RLP patients reported similar results. Marcia et al., observed a reduction of VAS from 8 to 3 after a year. Seventy percent of patients did not use analgesic [30]. In a study by Touraine et al., the baseline VAS fell from 7 to 3.8 and 2.6 after 4 and 12 weeks, respectively [29]. It is worth pointing out that chronic LBP can lead to central nervous system sensitization that can persist long after the source of pain was eliminated.

There are conservative and surgical treatment options in DDD. Except for the cases when a patient presents neurological deficits, bowel or bladder dysfunction, the choice is not straightforward and largely depends on the patient’s needs and expectations. It is worth emphasizing that DDD symptoms may disappear without any surgical intervention—the phenomenon thoroughly studied in lumbar disk herniation [47]. Chiu et al., estimated the spontaneous regression rate at 96% for disc sequestration, 70% for disc extrusion, 41% for disc protrusion, and 13% for disc bulging. Complete resolution of disc herniation was observed in 43% of cases of sequestrated discs and only 15% of extruded discs [48]. Takada et al., found that sequestrated discs were completely resolved after nine months, whereas extruded discs were only completely resolved after 12 months [49]. Much less is known about the DP natural history. Peng et al. performed a 4-year follow-up of 131 DP patients [50]. They found pain alleviation and lumbar function improvement in only 13% cases. Twelve percent of patients reported aggravated symptoms, and 67.2% did not experience any change in pain and disability. A slight improvement was observed in 7.6% cases. For the whole cohort, the pain and disability reductions were equal to 7.6% and 5.2%, respectively. Thus, in most cases, DP is chronic and persistent.

All our patients met the eligibility criteria for lumbar decompression surgery. We conformed to the national [51] and international [52] guidelines of radiculopathy treatment and performed GEI at least after 6 to 8 weeks of persistent symptoms. Taking into account the persistent nature of DP [50], we adopted the same time frame for the DP cohort. At successive follow-ups, the reduction of COMI with respect to baseline was observed in 77%, 74%, 84%, and 91% of DP and RLP cases. Thus, it is plausible that the improvement in the clinical condition may be partially attributed to the spontaneous regression. All our patients were fully aware of this possibility. They chose GEI primarily because of pain intolerance and the desire to return to work as quickly as possible.

The study’s primary limitation was the minimal duration of symptoms (6–8 weeks) which we used to qualify the patients for the procedure. We already mentioned that the regression rate for patients with disk protrusion is equal to 41%. Thus, long-term conservative treatment is certainly a viable option. The choice of therapeutic strategy is even more complicated for DP [53]. In particular, the evidence for drug therapy in chronic discogenic low back pain is limited.

## 5. Conclusions

The presented results indicate that ethanol gel’s efficacy is promising and similar in DP and RLP. The main advantages of this method, which is performed under local anesthesia in the outpatient setting, are its relatively low cost, simplicity, and because ethanol is injected, a theoretically lower risk of infection than other intradiscal treatments. As a minimally invasive technique, ethanol gel does not carry the risks of general anesthesia and postoperative complications. Quick pain alleviation observed in the high percentage of patients is important because it prevents central nervous system sensitization. Moreover, a potential failure does not exclude a patient from other surgical treatments. Future double-blind randomized controlled trials with follow-up to several years will be necessary to determine the efficacy and utility of ethanol gel compared to other intradiscal treatments.

## Figures and Tables

**Table 1:** Patient characteristics.

Parameter	Discogenic Pain	Radiculopathy
Sex	37 M, 8 F	12 M, 10 F
Age	44 (10)	50 (10)
Profession	WCW: 6, LW: 12, HW: 19, UN: 8	WCW: 3, LW: 11, HW: 2, UN: 6
Operated levels	L2/L3: 5, L3/L4: 9, L4/L5: 24 L5/L6: 3, L5/S1: 4	L3/L4: 6, L4/L5: 13 L5/L6: 1, L5/S1: 2

Legend: M—male; F—female; WCW—white-collar worker; LW—patients performing light physical work; HW—patients performing heavy physical work; UN—unemployed.

**Table 2:** Questionnaire results at baseline and subsequent follow-ups (F1–F4). Data are presented as mean (95%CI). A black lozenge indicates the follow–up values that were statistically different than those of baseline.

Parameter	Baseline	F1 (4–8 wks)	F2 (12–24 wks)	F3 (24–36 wks)	F4 (36–52 wks)
Discogenic pain					
COMI	6.40	4.61 ${}^{⧫}$	3.90 ${}^{⧫}$	3.16 ${}^{⧫}$	2.20 ${}^{⧫}$
	(5.88–6.92)	(3.80–5.43)	(2.81–4.99)	(2.37–3.95)	(1.53–2.87)
VA [cm]	6.33	4.03 ${}^{⧫}$	4.91	3.55 ${}^{⧫}$	2.97 ${}^{⧫}$
	(5.74–6.92)	(3.17–4.89)	(3.76–6.06)	(2.66–4.44)	(2.02–3.92)
VAS back [cm]	6.15	3.68 ${}^{⧫}$	3.30 ${}^{⧫}$	2.84 ${}^{⧫}$	1.80 ${}^{⧫}$
	(5.57–6.73)	(2.70–4.66)	(2.21–4.39)	(2.01–3.67)	(1.22–2.38)
VAS leg [cm]	3.57	2.76	2.62	2.26	1.60 ${}^{⧫}$
	(2.71–4.30)	(1.59–3.81)	(1.45–3.79)	(1.38–3.14)	(1.00–2.20)
Radiculopathy					
COMI	7.05	4.13 ${}^{⧫}$	3.62 ${}^{⧫}$	2.57 ${}^{⧫}$	3.68 ${}^{⧫}$
	(6.33–7.76)	(3.10–5.16)	(2.06–5.18)	(1.20–3.94)	(2.48–4.88)
VAS [cm]	6.77	4.00 ${}^{⧫}$	4.00 ${}^{⧫}$	2.33 ${}^{⧫}$	3.13 ${}^{⧫}$
	(5.89–7.65)	(2.84–5.16)	(2.19–5.81)	(1.35–3.31)	(1.79–4.47)
VAS back [cm]	5.14	3.17	2.76	1.55 ${}^{⧫}$	3.14 ${}^{⧫}$
	(3.98–6.30)	(2.14–4.20)	(1.19–4.33)	(0.68–2.42)	(2.14–4.14)
VAS leg [cm]	5.95	3.33 ${}^{⧫}$	2.24 ${}^{⧫}$	1.45 ${}^{⧫}$	2.57 ${}^{⧫}$
	(4.94–6.96)	(2.19–4.47)	(0.83–3.65)	(0.23–2.67)	(1.19–3.95)

**Table 3:** Anaglesic consumption in RLP and DP groups before and 6 months after procedure.

Consumption of Analgesics	Discogenic Pain		Radiculopathy	
	Pre	Post	Pre	Post
0 (no)	5	28	2	13
1 (once a week)	4	7	2	3
2 (several times a week)	5	1	4	0
3 (once a day)	8	0	2	0
4 (several times a day)	16	2	6	0

## Abbreviations

The following abbreviations are used in this manuscript:

AF	Annulus fibrosus
COMI	Core Outcome Measures Index
DDD	Degenerative Disc Disease
DP	Discogenic pain group
GBD	Global Burden of Disease Study
GEI	Gelified ethanol injection
IVD	Intervertebral disc
LBP	Low back pain
MRI	Magnetic Resonance Imaging
NP	Nucleus pulposus
RLP	Radiculopathy leg pain group
VAS	Visual Analogue Scale
VEP	Vertebral end-plate
